# Retroviral vectors and transposons for stable gene therapy: advances, current challenges and perspectives

**DOI:** 10.1186/s12967-016-1047-x

**Published:** 2016-10-12

**Authors:** José Eduardo Vargas, Leonardo Chicaybam, Renato Tetelbom Stein, Amilcar Tanuri, Andrés Delgado-Cañedo, Martin H. Bonamino

**Affiliations:** 1 Centro Infantil-Pontifícia Universidade Católica do Rio Grande do Sul-PUCRS, Porto Alegre, Brazil; 2 Programa de Carcinogênese Molecular, Instituto Nacional de Câncer (INCA), Rua Andre Cavalcanti 37/6º andar, Centro, Rio de Janeiro, 20231-050 Brazil; 3 Vice-presidência de Pesquisa e Laboratórios de Referência, Fundação Instituto Oswaldo Cruz, Rio de Janeiro, Brazil; 4 Universidade Federal do Rio de Janeiro, Rio de Janeiro, Brazil; 5 Grupo de estudo de Expressão Gênica de Eucariotas, Unipampa, Sao Gabriel, Brazil

**Keywords:** Gene therapy, Lentivectors, Transposons, Clinical trials

## Abstract

Gene therapy protocols require robust and long-term gene expression. For two decades, retrovirus family vectors have offered several attractive properties as stable gene-delivery vehicles. These vectors represent a technology with widespread use in basic biology and translational studies that require persistent gene expression for treatment of several monogenic diseases. Immunogenicity and insertional mutagenesis represent the main obstacles to a wider clinical use of these vectors. Efficient and safe non-viral vectors are emerging as a promising alternative and facilitate clinical gene therapy studies. Here, we present an updated review for beginners and expert readers on retro and lentiviruses and the latest generation of transposon vectors (sleeping beauty and piggyBac) used in stable gene transfer and gene therapy clinical trials. We discuss the potential advantages and disadvantages of these systems such as cellular responses (immunogenicity or genome modification of the target cell) following exogenous DNA integration. Additionally, we discuss potential implications of these genome modification tools in gene therapy and other basic and applied science contexts.

**Electronic supplementary material:**

The online version of this article (doi:10.1186/s12967-016-1047-x) contains supplementary material, which is available to authorized users.

## Background

Genetic modification has played a major role in cell biology studies aiming to describe cellular mechanisms and pathophysiological processes. The ability to express foreign proteins and non-coding RNAs, to knock down protein expression by shRNA and, more recently, to edit the genome of cells allowed the elucidation of several genetic and biochemical systems in living organisms by interfering with their physiology. Recently, these technologies are being widely explored due to their potential for gene therapy.

Adding a new genetic unit can deeply impact the biology of individual cells and the entire organism. While shRNA and genetic edition of DNA usually require transient or permanent expression for their effects to take place, the permanent expression from a transgenic unit usually requires it to be integrated in the genetic material of the organism so it can be passed from the originally modified cells to the daughter cells.

The deeper knowledge of biological signaling circuits and networks led to the development of a whole new field of synthetic biology, in which single or multiple genes are transferred to cells, ascribing new functions and, ultimately, potentially impacting the whole metabolism of multicellular organisms. As examples of such new applications, yeasts have been recently modified to build a whole biosynthesis pathway for bioactive molecules by adding 23 new genes [1] and lymphocytes are being modified with multiple genes in order to sense outside stimuli integrating the signals in conditional [2] or logic gates based approaches [3]. The nature of outside signals can vary from proteins or ligands, to metabolites or even light, as recently demonstrated in approaches based on optogenetics activation of genetic units [4].

While complex modifications of cells for therapeutic use are still under development (e.g. genome editing), straightforward approaches, such as adding a genetic unit to human cells, are currently being investigated in several diseases as a therapeutic approach with outstanding results in some contexts.

The proper choice of the tool to be used in order to transfer the genetic cassettes to eukaryotic (and especially to mammalian) cells varies depending on the size, number and even complexity of the genetic unit(s) to be transferred. The genetic modification of cell lines for cell biology studies or its application in biotechnological processes has different requirements, such as transfection or transduction efficiency and clonal expansion, if compared to human cell modifications for therapeutic purposes.

In the current review, we focus mainly in the available systems and efforts to genetically modify cells through the use of tools such as gammaretro and lentiviral vectors and transposons that currently or potentially accomplish safety and efficiency requirements for clinical applications in gene therapy protocols.

## Gene therapy

Gene therapy is defined as a set of strategies that modify the expression of an individual’s genes or to correct abnormal genes of a defective cell to reestablish the normal function. In diseases related to recessive gene defects, complementing the genome with a functional sequence can often revert the phenotype even if mutated copies remain in the cell. In pathologies linked to dominant mutant copies of a gene, knocking out, knocking down or replacing the mutated copy may be mandatory. In this sense, viral and non-viral systems were designed for gene transfer, where each system has advantages or disadvantages in terms of clinical gene therapy applications and protocol developments. Considering viral vector-based approaches, retroviruses have an intrinsic capacity to integrate into the target cell genome and were shown to be efficient in transducing mammalian cells both in vitro and in vivo [5–11]. The most popular members used for gene therapy are the retrovirus vectors based on murine Moloney leukemia virus (MLV) and human immunodeficiency virus type 1 (HIV-1). Non-specific integration of the viral DNA in the host genome can cause gene disruption, inducing modifications of open reading frames of native genes or abnormal expression of genes nearby the insertion site by interfering with enhancer activities. These interferences can alter critical cellular functions such as cell cycle control, ultimately inducing oncogenesis [12].

The overall goal of treating chronic diseases originating from genetic deficiencies can be potentially limited by the necessity of re-administrating the vector to treat patients throughout their lives. Another difficulty is based on technical principles, such as the need for extensive purification of the products to be infused to avoid or reduce immunologic events triggered by the vector, as well as costs associated with their production and manipulation. Advantages of non-viral vectors include their easy manipulation and the relatively low cost to produce sufficient vector quantities to treat a patient, stability during storage and low immunogenicity [13].

In this context, DNA transposons have been shown to be an attractive choice for gene therapy. Here we review recent advances in the design of the modified piggyBac (PB) and Sleeping Beauty (SB) DNA transposons, which are highly efficient in mediating the stable integration and expression of transgenes in human cells and mice. Thus, we review recent progress in the molecular biology of these stable gene-transfer tools, discussing the state-of-the-art in the application of transposable elements for therapeutic gene transfer.

## Retroviruses and stable gene therapy

Viruses are the most highly evolved natural vectors for delivering foreign genetic material into cells. This feature has led to extensive strategies to engineer recombinant viral vectors for the delivery of therapeutic genes into tissues/cells. The majority of viruses elicit a host immune response [14–16]. For this reason, low immunogenicity is fundamental for successful gene therapy approaches using viral vectors.

Retroviridae are classified as Class VI viruses based on the Baltimore Classification of Viruses, this is due to their genome being plus sense RNA and having a DNA intermediate in its life cycle. According the International Committee on Taxonomy of Viruses (ICTV), 2015 release, the family retroviridae consists of two subfamilies, seven genera, and fifty-three species. Murine oncoretroviral vectors are derived from murine leukemia virus belonging to the gammaretroviral genus. On the other hand, lentiviral vectors are derived from human immunodeficiency viruses type-1 (HIV-1), lentivirus genus. Retroviridae family viruses are characterized by their RNA genome, which is retrotranscribed to DNA by the reverse transcriptase enzyme. This DNA is integrated into the host cell genome, allowing long-term viral gene expression by the infected cells and their progeny. In the past two decades, these proprieties have turned retroviral vectors into an attractive system for use as cargo for foreign gene expression in mammalian cells. During retroviral construction, the genes necessary for viral infection are provided in *trans*, being expressed in different plasmids. Thus, it is possible to generate a replication-defective virus that does not produce pathogenic effects in the cells, making these systems potentially safe. For these reasons, retroviral systems are efficiently employed in gene transfer or gene therapy protocols. Vectors based on HIV-1 or MLV have been used in a great number of preclinical experiments and clinical trials in the last two decades.

### Functional retroviral genome: viral infection at the service of biotechnology and therapy

**Fig. 1:**
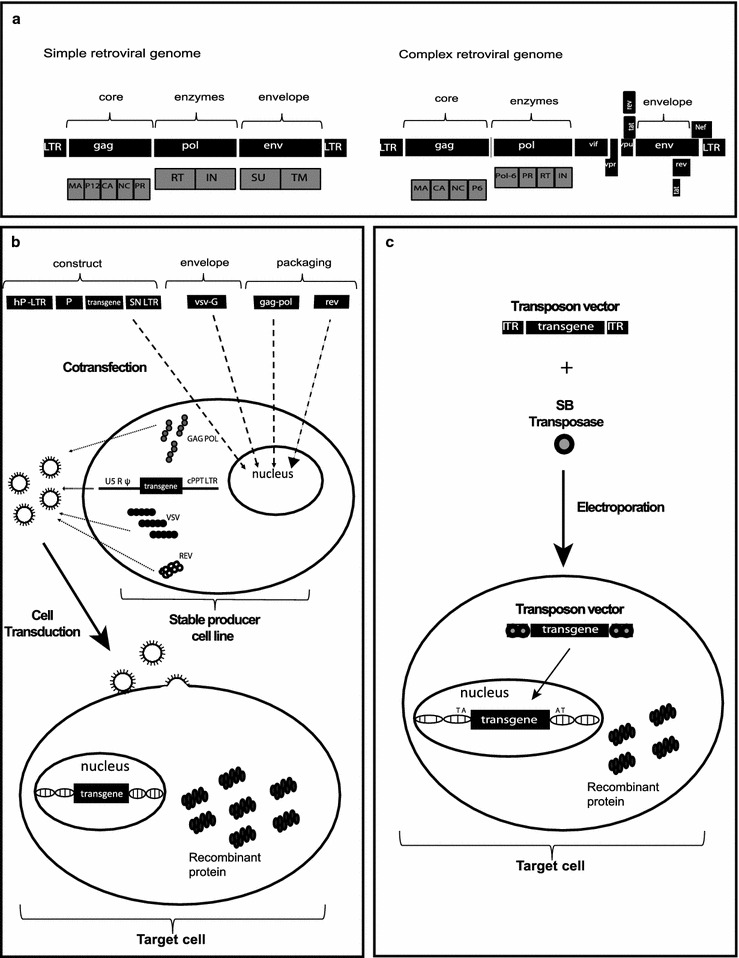
Stable gene expression systems. **a** Representation of simple (e.g. MLV) and complex (e.g. HIV-1) retroviral genomes. **b** Lentiviral production of 3rd generation vectors and cell transduction. Plasmids containing expression constructs of genetic elements required for packaging (gag-pol, rev and VSV-G, a gene encoding the fusogenic envelope G glycoprotein of the vesicular stomatitis virus) and a plasmid of interest comprised of a chimeric 5′ LTR (long terminal repeat) fused to a heterologous promoter (hP), a promoter (P) to control transgene expression and 3′ Self-inactivating (SIN) LTR are co-transfected together into a producer cell line. After viral production, transduction of lentivector is performed on the target cell. **c** Cut-and-paste mechanism of *SB* transposons, where a transposon in a plasmid and a transposase binds to two inverted terminal repeats (ITRs) of the transposon, and precisely cuts the transposon out of the plasmid, inserting the transposon into DNA of the target cell. SB transposons, integrate into TA dinucleotide base pairs, which are duplicated on each end of the insertion site

The *Retroviridae* family comprises enveloped non-icosahedral viruses with a genome composed of two copies of single-stranded RNA. The genome is non-segmented with positive polarity, ranging from 7 to 12 kb [17]. Three main open reading frames (ORFs) are essential to produce structural proteins and enzymes for viral metabolism (gag, pol, and env). In simple viruses such as MLV, these three ORFs are sufficient for viral replication and pathogenesis [18, 19]; lentiviruses, in contrast, are complex retroviruses that require additional genes for their physiopathology (Fig. 1).

Amongst viral genes, *Gag* encodes structural glycoproteins and is required for the assembly of non-infectious and immature viral-like particles; *pol* encodes enzymes necessary for viral replication and integration into the host cell genome (a protease, reverse transcriptase, and integrase). Another fundamental gene is *env,* which produces proteins embedded in the viral membrane that enable viral attachment to cellular receptors and fusion with target cells, determining the tropism of these viruses [20–22]. In complex human retroviruses, on the other hand, as exemplified by lentiviruses such as human immunodeficiency virus (HIV), additional proteins are necessary for the efficient expression of viral genes and for viral replication. These include *tat* (retrotranscriptional regulator [23]), *rev* (RNA transporter [24]), and *nef* (immunomodulator), *vpr (cDNA* transport to nucleous [25]*), vif* (APOBEC degradation [26]) and *vpu* (theterin degradation). All the required genes for the lentivirus cycle are depicted in Fig. 1a. Reverse transcription and integration require LTR (long terminal repeat) sequences in the extremities of the viral genome. The integrase enzyme recognizes the terminal LTRs in the double-stranded DNA molecules previously synthesized by reverse transcriptase. After integration, the cellular RNA polymerase II transcribes the retroviral genes [27]. The 5′ LTR sequence includes a strong promoter region containing several *cis* elements for transcription-factor binding and a highly active initiator sequence. An additional enhancer region is composed of two NFkB-binding motifs [28] that act to increase gene expression based on the binding of NFkB and NFAT. The 3′ LTR acts as the termination and polyadenylation site for all viral ORFs [29]. The genomic RNAs are packaged into viral particles at the cell membrane. Packaging of the genomic transcripts requires the ψ sequence that lies downstream of the 5′ LTR [30], assuring that RNA lacking ψ will not be packaged.

For gene therapy purposes, the retrovirus backbone is depleted of all viral ORFs. In the region between the LTRs is added a therapeutic sequence, keeping the sequences required for the essential steps of vector production such as the psi packaging sequence and the long terminal repeats (LTRs) that are necessary to inserting the viral genome into the host DNA. These are called *cis*-acting elements because they need to be in the genomic RNA. *Trans*-acting elements are viral elements that can be encoded on a different RNA molecule. It is important to note that biotechnological tools derived from retroviral genome manipulation are designed to avoid the generation of replication-competent retroviruses that can arise from the recombination of cis and trans elements (packaging plasmids), thereby restoring a functional retrovirus genome carrying all the information required for functional replication (Fig. 1b).

There are excellent reviews elsewhere that carefully describe the process of generating different retro and lentivectors, including different versions of the constructs designed as 1st, 2nd, 3rd and 4th generation lentivirus vectors according to the number of plasmids and entire or truncated viral sequences used to encode essential components of viral genome with increased safety. We direct the reader there for details of self-inactivating (SIN) construct generation and characterization [31–33].

### Clinical trials

There are currently over 417 human clinical trials involving retroviral gene therapy registered in the Journal of Gene Medicine database (http://www.abedia.com/wiley/vectors.php, accessed in July, 2016). The first successful gene therapy protocol occurred in the 1990s. In that protocol, two patients with severe combined immunodeficiency (SCID) due to adenosine deaminase (ADA) deficiency were treated with a retroviral vector carrying the ADA coding sequence under the transcriptional control of the promoter/enhancers of the long terminal repeat of the MLV. ADA disease is characterized by defective T and natural killer cell maturations as well as low B cell function, causing recurrent infections. In this pioneer trial, one of the treated patients recovered cell counts and function, showing no adverse effects after 4 years. The response was more limited in the second patient primarily due to lower transduction efficacy; however, other causes could have contribute to this low efficiency such as immune responses against the retroviral envelope or the fetal calf serum used during ex vivo cell expansion [34]. In the same decade, two additional clinical trials observed normalization of T lymphocyte counts in patients treated with murine γ-retroviral vectors [35, 36]. However, in these two trials patients received simultaneously enzymatic replacement, impairing the unequivocal evaluation of the direct effect of gene therapy. From 2000s to today, improvements of clinical trials using γ-retroviral vector carrying a functional copy of ADA in autologous CD34+ cells were performed for ADA-SCID therapy [37–41]. Follow-up studies showed gene correction in multiple cell lineages, leading to the expression of normal ADA levels and restoration of immune competence. It is further encouraging that more than 40 ADA-SCID patients were treated with these vectors without genotoxic consequences [42].

The results of the first gene therapy trial for ADA-SCID increased optimism regarding an effective treatment based on gene transfer for several monogenic disorders using other viral vectors. The excellent results led ADA gene therapy to be ultimately approved for commercialization in Europe in 2016. However, in 1999, the death of one patient enrolled in a clinical trial designed to treat ornithine transcarbamylase deficiency using adenoviral vectors put the whole field on hold until regulatory agencies released the ongoing trials [43]. Despite this negative event, it is necessary to clarify that it did not occurred due to an integrative event, because contrarily to retroviral vectors, adenoviral vectors are not integrative [44]. Shortly after this adverse event, another trial showed the darker aspects of gene therapy protocols applying integrative vectors in a clinical trial for X-linked severe combined immunodeficiency (X-SCID). This disease is caused by the lack of the common gamma chain (γc), which is present in several interleukin receptors and indispensable for T cell development. Between 1999 and 2006, patients were enrolled in several gene therapy protocols aiming to restore γc expression on CD34+ cells. Seventeen of the twenty treated participants were alive and displayed nearly full correction of their T-cell deficiency, presenting genetically modified T cells, when evaluated between 5 and 12 years after the gene therapy procedure [45]. Five participants developed T-cell leukemia 3–6 years from gene therapy. Four of the patients were treated for leukemia and achieved complete remission, but one leukemia patient died of refractory disease [46, 47]. Vector integration in these patients identified insertions near the LMO2 proto-oncogene promoter, leading to aberrant transcription of LMO2 [47–50]. LMO2 transcription disruption seems not to be the only hit leading to leukemogenesis since NOTCH1 mutations and deletions of some tumor suppression genes were also reported [51]. These results were enough to halt gene therapy trials using MLV-based vectors in various countries, such as France, England and the United States. In addition, the American National Institutes of Health (NIH) suggested stopping active protocols using MLV for gene therapy.

Despite these adverse events, the clinical use of chimeric antigen receptors or suicide genes on T lymphocytes has been repeatedly reported with no adverse effects as a consequence of gene transfer [52], suggesting that T cells are safe populations for MLV-based gene transfer [53]. Suicide-genes for T cell elimination on demand after the transplantation of transduced T cell for the treatment of disease such as cancer and graft versus host disease were also developed and ultimately clinically applied. Among these genes, herpes simplex virus thymidine kinase (HSV-TK) is still accepted as a reference strategy. HSV-TK-based cell elimination results from the phosphorylation of a prodrug by thymidine kinase, which is converted to a toxic drug that interrupts DNA elongation and causes apoptosis [54]. This strategy is currently under investigation in a phase III clinical trial in patients undergoing haploidentical stem cell transplantation [55, 56]. Other strategies to eliminate transduced cells include the inducible caspase 9 (iCasp9) switch, carrying a protein consisting of the fusion of the human caspase 9 and a modified human FK-binding protein, which allows conditional dimerization [57]. Upon exposure to a synthetic dimerizing drug (AP1903), the inducible iCasp9 becomes activated and leads to the rapid death of cells expressing this genetic construct. This approach showed outstanding results in clinical trials for the elimination of T lymphocytes causing graft versus host disease [58].

However, it is important to note that MLV vectors have the biological disadvantage that they are unable to efficiently transduce non-dividing or slowly dividing cells. As a result, MLV vectors were gradually replaced by lentiviral vectors based on HIV-1, which can integrate in the host genome of non-dividing cells nearly as well as in dividing cells [59]. Lentiviral vectors finally entered clinical trials a few years ago, and several gene therapy protocols are currently underway with impressive results around the world. In 2001, the first human subject was treated with lentiviral vectors. Autologous CD4+ cells from HIV + patients were transduced with a lentiviral vector based on HIV-1, containing antisense sequences against the HIV-1 envelope gene [60]. Thereafter, gene therapy with lentivectors was extended to the pre-clinical study of several monogenic diseases, such as hemophilia [61], a X-linked bleeding disorder caused by mutations in Factor VIII or Factor IX genes and; metachromatic leukodystrophy [62], caused by arylsulfatase A deficiency. In 2007 the firsts clinical trials of monogenic diseases, adrenoleukodystrophy and beta-thalasemia, took place [63–65]. More recently, gene transfer via lentiviral vectors was shown to revert the disease state in the long term in patients of metachromatic leukodystrophy and Wiskott-Aldrich syndrome [66, 67]. These studies indicate that lentiviral-based gene therapy is a safe and effective approach to treat distinct diseases [65]. Furthermore, concerning ADA-SCID therapy, it is important to highlight that the trials using retroviral vectors presented similar survival rates to those achieved in hematopoietic stem cell (HSC) transplantation in patients undergoing an HLA-matched donor transplant [68]. Until now, there is only one clinical study reporting negative side effects due to insertional mutagenesis of lentivirus based vectors (as detailed in 4.3.1) and, although some clonal patterns in hematopoietic reconstitutions were suggested, this clonal skewing with transduced cells led to no evident clinical implications [69, 70].

Recently, lentivirus-based vectors were also applied in clinical trials to transfer chimeric antigen receptor (CAR) genes to T lymphocytes, leading to impressive leukemia elimination in patients treated with gene-modified T cells [71], reinforcing the concept that T lymphocytes are safe targets for gene therapy.

According to the Journal of Gene Medicine database, there are currently 114 clinical protocols registered to treat several diseases (including cancer) around the world using lentiviral vectors, representing about 21 % of all retroviral gene therapy protocols, demonstrating the wide applicability of this vector backbone. In this sense, we present a detailed table including major technical aspects of these 114 protocols (Additional file 1: Table S1). Interestingly, handling of patient’s autologous cells is registered in over 90 % of all clinical trials (Additional file 1: Table S1). Currently, nearly 46 % of these clinical trials are dedicated to cancer treatment, 14 % to HIV and the remaining percentage to monogenic diseases. Several reports describe clinical efficacy of gene therapy protocols based on lentiviral systems for human cancer and HIV treatment [71–74]. Their efficacy at promoting potent anti-tumor immune responses certainly relies in their capability to ensure a persistent expression of the desired transgene, such as the molecules designed to efficiently boost T cell effector functions. Other retro and lentiviral manipulations of T lymphocytes are under development to increase antitumor T cell function and target cell specificity as recently reviewed in Chicaybam and Bonamino [2].

### Integrating vectors: drawbacks and potential pitfalls

Stable retroviral expression is the final aim of gene therapy protocols using this powerful therapeutic tool but can potentially produce two primary problems that influence the vector suitability for specific therapeutic applications: insertion mutagenesis and/or the destruction of transduced cells by the immune system. Here, we review the most relevant data reported in the literature describing the deleterious effects of provirus integration-mediated genotoxicity.

#### Retroviral integration pattern, a potential problem for gene therapy

Provirus integration for MLVs is mainly described in promoter and enhancer sequences of the target cell genome, while lentiviral vectors preferentially integrate throughout gene sequences [75–77]. In both cases, provirus integration can potentially disrupt the gene structure altering its transcription or function, ultimately leading to oncogenesis. Insertional mutagenesis was reported in a β-thalassemia patient who was treated with a self-inactivating (SIN) HIV-1-based vector containing the β-globin gene controlled by its wild-type promoter. The patient was treated with autologous CD34+ cells transduced with a lentiviral vector [65]. Clonal population analysis demonstrated a bias for one hematopoietic clone derived from transduced cells with the proviral cassette integrated into the *HMGA2* proto-oncogene sequence causing a benign cell expansion. Interestingly, HMGA2 was overexpressed in myeloid cells, but the deregulation was not observed in granulocyte-monocyte cells sharing the same vector integration pattern. Thus, studies to understand cell-type-dependent deregulation could help to develop improved methods for ex vivo cell handling, which promote an efficient monitoring of patients to obtain a safe gene therapy.

Due to the necessity of tracking the vector insertional pattern during the treatment, several clinical trials using lentiviral-vector-based HSC, analyzed transduced clones in the reconstituted haematopoiesis. The results showed a cell population without the emergence of dominant clones capable of promoting the development of neoplasic events. An in-depth molecular analysis of the reconstituted haematopoiesis is systematically realized in subjects transplanted with hematopoietic cells transduced with retroviral vectors, helping to develop the first reliable comparative assessment of vector-induced events in patients [34, 61, 64, 66, 67, 78]. Tracking clonal activity in the reconstituted haematopoiesis is a necessary step to guarantee the safety of gene therapy protocols, a point reviewed and discussed in detail by Naldini et al. [69]. Investigators developed novel strategies aiming to avoid, or at least reduce, incidental gene disruptions refining these vectors through the creation of retroviral vectors containing insulators that increase the autonomy between nearby transcriptional units by blocking the interaction between enhancer and promoters or by suppressing the spread of heterochromatin. Most of them are derived from a cHS4 element of the chicken β-globin locus [reviewed in [79]. Some in vitro studies reported a reduction of the transforming potential to the background levels when lenti or retrovectors containing insulators are used [80–82], but one study performed by transducing Jurkat cells suggests that insulators are not sufficient to avoid proliferative or survival advantage conferred by some integration events leading to clonal dominance events [83]. On the other hand, mutation in the insulators can affect its function [65]. It is important to note that these side effects do not exclude hematopoietic cells as targets for gene therapy. In the Northstar Study (HGB-204), HGB-205 and HGB-206 clinical protocol researchers evaluated the use of a lentiviral vectors that transport an engineered β^A−T87Q^-globin gene (LentiGlobin BB305 Drug Product) into patients hematopoietic autologous CD34+ cells. Preliminary results of these trials indicated that patients with β-thalassemia major after hematopoietic cell transplantation with the lentiviral product experienced consistent β^A−T87Q^-globin production, leading to transfusion Independence for at least 15 months [84]. After a median follow-up of 198 days, no clonal dominance was detected in these patients [85]. Furthermore, one subject with severe SCD, treated with LentiGlobin BB305 lentiviral vector, remains free of SCD-related events by producing approximately 51 and 49 % of anti-sickling globins and HbS, respectively [84].

It is clear that specific studies are also required to define the mechanism of action of retroviral pre-integration complexes in order to develop alternative strategies, avoiding deleterious effects of insertional mutagenesis. This will hopefully allow the design of modified integrases with the ability to integrate DNA only in a specific genome sequence.

Strategies to fuse the viral integrase with specific sequences of DNA-binding domains obtained from bacteria (LexA) [86, 87] or mammalian (zinc finger protein zif268) [88] have been developed. However, these attempts showed limited success without major modification in the genome integration pattern when compared to wild-type integrases.

#### Immune system and lentivectors

As discussed before, the appropriated level of transgene expression is essential to gene therapy approaches in protocols using either lentiviral vectors or non-viral methods. The immune system is a natural barrier that can influence transgene expression. Controversial information about immunologic responses against transduced cells has been reported in the literature. For instance, in an in vivo murine model, the expression of coagulation factor IX, mediated by a lentiviral integrase-defective vector in hepatocytes elicited tolerance to the transgene without induction of neutralizing antibodies [89]. In contrast, in a similar model, hepatic lentiviral administration induced rapid and transient IFN-α/β response and promoted functional cytotoxic T lymphocyte (CTL) responses [90].

Several reports in mouse models suggest that the elimination of transduced cells is mediated by CTL responses. These responses can be attenuated by tissue-specific promoters present in the backbone of the lentiviral vector or exacerbated by viral envelope proteins that can be highly immunogenic [91].

Despite the reported immune responses to the transgene that can limit its expression, lentiviral vectors induce very limited immune and inflammatory responses associated with the vector itself [9, 19].

## Transposon-transposase vector systems

Despite being one of the most used gene-transfer systems, viral vectors have important hurdles to overcome regarding their clinical application, such as large-scale vector production and careful biosafety characterization, which have major impacts on the costs of clinical-grade vector stock production. In recent years, non-viral DNA transposon based-systems have emerged as a potential tool that might overcome some of these limitations.

DNA transposons are mobile genetic elements shown to be present in all animal phyla [89]. There are thousands of families of these elements, and the majority of them have a transposase gene flanked by inverted terminal repeats (ITRs). The transposase, through a “cut and paste” mechanism, recognizes the ITRs, excises the transposon and integrates it in another site of the genome. These elements have important roles in the evolution of genomes, constituting a considerable fraction of host DNA in several species [92].

Due to its integration capacity and non-viral nature, some of these transposons were adapted for use in gene therapy protocols. To achieve efficient and safe use, the transposons were split in two plasmids that are co-transfected in the cell, one containing the sequence encoding the transposase enzyme and the other containing an expression cassette flanked ITRs (Fig. 1c).

This design has the following advantages when compared to viral vectors:

- Decreased production costs: plasmid production under Good Manufacturing Practices (GMP) conditions is much faster and cheaper than viral vector production. Moreover, there are no cumbersome quality-assurance procedures, such as titration of vectors and testing for replication-competent virus.
- Increased biosafety: because it involves only the manipulation of plasmids, it can be easily performed in a biosafety level 1/2 laboratory with basic equipment, without requiring complex biohazard contention procedures.
- Low immunogenicity: in vivo applications of VSV-G pseudotyped vectors and adenoviral vectors are often limited by immune recognition of viral proteins, which may not occur when using plasmid-based vectors.

However, despite these advantages, DNA transposon-based vectors are essentially gene-inserting tools that still need assistance for efficient cellular uptake. Activity may therefore vary depending on transfection method selected, cell type, and plasmid size. Moreover, it is important to note that these vectors have been largely used in the preclinical setting, and clinical trials are underway to evaluate their efficacy, safety and presumed advantages.

Several transposon systems have been developed, allowing the application of this technology in different model organisms, such as *Tol2* for zebrafish [93, 94]. In this review, we focus on the two main systems used in mammalian cells, *Sleeping Beauty* and *piggybac,* and we discuss their efficiency, improvements and applications for clinical trials.

### Sleeping beauty—SB

For many years, the use of DNA transposons as a gene-transfer system was hampered by the lack of active elements in mammalian genomes. In 1997, pioneering work by Ivics and colleagues [95] developed the *sleeping beauty* (SB) transposon from inactive copies of *Tc1/mariner*-like elements found in several fish genomes. This transposon was shown to be active in tissues of different vertebrate species, including humans, and showed no signs of endogenous transposon cross mobilization. It has modest cargo capacity, allowing the efficient transposition of genes up to 6 kb, which is sufficient for most applications. Beyond this limit, the transposition rates rapidly decay [96], although recent studies using improved versions of the transposon vectors (T2 and sandwich versions) showed efficient integration of transgenes of up to 18 kb [97]. It has been shown that this system is more efficient under limiting quantities of transposon DNA, which occurs in hard-to-transfect cells like CD34+ hematopoietic cells [98]. A disadvantage of the SB system is the overproduction inhibition phenomenon, achieving less transposition at higher transposase concentration, which is thought to occur due to misfolded or aggregation of this enzyme [99]. Thus, careful titration of the transposase is needed to determine the optimal transposon/transposase ratio to be used [100]. Importantly, the delivery of SB transposase in the form of RNA was shown to be much less toxic when transient expression at a low level using mRNA transduction approach was used [101].

Despite early in vivo applications of SB showing efficient integration of the transgene in hepatocytes [102, 103], the transposition activity of SB was low, limiting the applicability of this approach. The development of hyperactive SB mutants [96, 104–106] increased transposition rates up to 100-fold when compared to wild-type SB, leading to efficiencies comparable to retro- and lentiviral transduction in some applications [107]. The use of hyperactive mutants in vivo resulted in long-term expression of the transgene and phenotypic corrections in models of mucopolysaccharidosis type I [108] and hemophilia [109] for example. The generation of sets of transposon plasmids containing different fluorescent proteins and selection markers improved the flexibility of the system, increasing the possible applications of this system [110].

Moreover, the SB system is being successfully used for the ex vivo transfer of TCR or CAR genes for the generation of antitumoral T lymphocytes [107, 111, 112]. These T cells maintain high expression of the 19BBz CAR after in vitro expansion [113] and showed antitumor activity in preclinical models of leukemia [114, 115]. It is important to note that compared to other transposon systems, such as *piggyBac* and *Tol2*, the SB system displays a safer integration profile, integrating in TA dinucleotides in a close to random pattern. This is in sharp contrast to *piggyBac* and *Tol2*, which showed integration profiles similar to retroviral vectors, with integration sites near transcription start sites [116]. Furthermore, the ITRs of transposon vectors have a low promoter/enhancer activity similar to the SIN LTR of retro/lentivirus vectors, minimizing the risk of promoter/enhancer interference [117]. These properties prompted investigators to start a clinical trial using SB-modified T cell therapy for the treatment of B-cell malignancies [118]. These results were recently described and showed that the use of SB-modified CAR T cells is safe when infused after autologous or allogeneic hematopoietic stem cell transplantation as an adjuvant therapy. These cells persisted for an average of 201 or 51 days in the autologous or allogeneic setting respectively, and patients showed progression-free survival rates that were improved when compared to historical data [119].

Finally, the SB system has successfully been used for transgenesis in mice, rats and rabbits, achieving better efficiency than pronuclear injection and lentivirus-based protocols [120]. SB-mediated transgenesis was shown to be less prone to mosaicism and gene silencing when compared to the methods cited above and allows the generation of founders harboring a single copy of the transgene by titration of the transposase. Similar results were obtained in large animals, like pigs [121] and cattle [122]. This system also showed superior efficiency in the in vitro genetic modification of human CD34+ hematopoietic stem cells compared to the *piggyBac* system, making SB a reliable alternative to lentivirus vectors that are routinely used in this setting [123]. A recent publication reported an elegant reprogramming strategy to generate transgene-free iPS cells based on SB constructs [124].

The major challenge faced by transposable systems such as SB or other transposons is the delivery of plasmids to the target cells. Several strategies can be used for this purpose, including in vitro [112] and in vivo [125] electroporation of target cells and hydrodynamic injections [103]. Viral delivery of transposase and transposon using adenovirus [126] and non-integrating retrovirus [101] or lentivirus [127] can bypass these hurdles, providing efficient delivery in vitro and in vivo for different types of cells. Recently, the delivery of SB transposon and transposase in the form of DNA minicircle vectors showed increased transposition rates in cell lines [128] and, when used in conjunction with methotrexate selection, allowed efficient stable expression of up to three different transgenes [129], widening the potential applications of the technology. Updated clinical trials protocols using SB system are showed in the Additional file 2: Table S2.

### piggyBac (PB)

Although pioneered by SB, the transposon toolbox was expanded and developed with the discovery of other transposable elements. The piggyBac transposon, isolated from the cabbage looper moth *Trichoplusia ni* [130], showed high transposition activity in different mammalian cells [131, 132]. The most common setup is, as in SB, the use of a two-plasmid system, one containing the expression cassette flanked by ITRs and the other coding the PB transposase. This system is capable of delivering large inserts (up to 14 kb) without a significant loss of efficiency [131], and recent work showed that genomic regions up to 100 kb can be transposed, allowing a more physiological regulation of gene expression by transfer of entire regulatory regions [133]. The transposition occurs in TTAA sites and, unlike SB, which has a mobilization footprint of 3 bp consisting of the terminal three base pairs of the transposon flanked by TA dinucleotides [100, 134], the PB transposase completely restores the integration site upon mobilization [130, 135]. The PB transposase was shown to be tolerant to engineering, such as the development of an inducible system by fusion with ERT2 (making it responsive to 4-hydroxytamoxifen) [136], and the generation of a hyperactive version, called mPB, with a 17-fold increase in excision and ninefold increase in integration [137]. Moreover, a recent paper developed an excision-competent but integration-defective PB transposase, allowing excision of the transgene without reintegration in other genomic loci [138]. Despite these advances, the PB transposase is also susceptible to overproduction inhibition, even in in vivo models, although it has been rarely reported and is still a matter of debate [139].

These features render mPB system very useful for applications where transient expression of genes is sufficient, such as the generation of transgene-free iPS cells [140–142]. Importantly, when using mPB, the reprogramming efficiency is comparable to protocols using retroviral vectors, making PB one of the most useful transposon systems for this type of application [143]. It is also effective in BAC transgenesis, where the transfer of large sequences is needed [144]. Moreover, the PB system is the only one that showed activity in parasites, and the generation of transgenic *Schistosoma mansoni* has been recently reported [145].

However, the use of PB system for other applications may encounter some important obstacles. The 5´ITR of PB was shown to have transcriptional activity that might interfere with nearby promoters [136]. The PB system also showed an integration pattern similar to retroviral vectors, integrating mainly in transcription start sites and transcription units, raising concerns about the long-term safety of these vectors [116, 146]. PB may also integrates in sites other than TTAA nucleotides at low frequencies (~2 %), but these insertions can cause mismatches that could potentially generate point mutations in the genome [147]. This pattern of integration is useful as a gene-discovery tool using gene-trap cassettes [148]. Currently, there are no clinical trials underway using this system, and the described properties may limit its application in a clinical setting, where a safer integration pattern is required. A recent report showed that mutations in the transposase to increase function may also change the integration pattern [149] and it remains to be seen if this property can be used to purposely alter the transgene integration profile.

Despite this limitations, modifications of clinically relevant cells are being developed for a variety of human diseases, i.e., hESCs [47], hiPSCs [142, 150], HSCs [98] and human T lymphocytes. T lymphocytes are an attractive target for adoptive immunotherapy for cancer. Human T cells that were modified using piggyBac-transposons killed CD19-expressing human lymphoma cell lines, showing a functional activity of this transposon in this cellular model [151]. In addition, stable transgene expression using PB showed an efficiency of up to 40 % without selection in primary T cells in culture [152]. Moreover, integration site mapping showed that this transposon did not integrate into or near known proto-oncogenes [152]. According, PB would seem to be a promising nonviral system for cancer immunotherapy based on T cell modification in a future clinical trial.

### Safety issues of transposon vectors

The results obtained in preclinical studies using transposon vectors prompted researches to evaluate these systems in clinical trials. Given the safer integration profile [153] when compared to retrovirus [154], lentivirus [155] and PB [116], SB is currently being tested in ten clinical trials of T cell immunotherapy (Additional file 2: Table S2). As a high copy number is not desired due to the risk insertional mutagenesis, a transposase with an intermediate activity (SB11) is being used in these studies. Besides insertional mutagenesis, the main risk associated with SB-mediated gene therapy is the remobilization of the inserted transposon. This case results from the theoretically possible residual activity of transposase due to the unlikely integration of transposase-encoding plasmid, causing the inserted transposon to “jump” to a new genomic location and induce new alterations. However, due to the autoregulated activity of DNA transposons, the remobilization requires optimal rates of transposon/transposase, being highly inefficient in low or high concentrations of transposase [156]. The footprint of 3–5 bp leaved by SB remobilization could also induce a frameshift if the transposon was inserted in an exon, but the probability of this event is very low [157]. To exclude these possibilities, the transposase can be transfected in the form of RNA, which is also less toxic to the cells [107]. In above mentioned clinical trials using SB11 transposase, the modified T cells are evaluated for the presence of residual SB11 plasmid and for TCR clonality before infusion into patients to safeguard SB remobilization [158].

Although SB transposon-based gene transfer is considered a safer tool due to its integration pattern, all the mentioned tools lack specificity in sequence integration. As so, one caveat of the unparalleled efficiency of these tools is the inability to direct transgene integration into the host cell genome. Enabling integration in the genome in a sequence-based fashion opens the possibility for new genes to be integrated in safe harbors, regions where no relevant genes were mapped, increasing the safety profile of these already extremely useful gene modification tools. Recent studies have approached this by combining SB [159] or PB [160] transposases with ZF (zinc finger) or TALE DNA-binding domains respectively, directing the integration of transposon to pre-determined regions of the genome.

## Conclusions and perspectives

The last few decades witnessed a revolution in the development and application of gene therapy. There is currently no doubt that gene modification approaches have turned into a valuable biotechnology and therapeutic tool. New and safer vector designs along with a better comprehension of vectors biology led to successful utilization of these valuable tools in several clinical contexts now. The success of retro and lentivirus-based gene therapies helped to turn gene therapy into a solid and flourishing field. Non-viral integrative vectors, such as transposons, have the potential to extend this success story, hopefully making gene therapy approaches more straightforward, simple and cost-effective.

The newly developed genome-editing technologies such as zinc finger nucleases (ZFNs), transcription activator-like effector nucleases (TALENs) and Clustered regularly-interspaced short palindromic repeats (CRISPRs) represent the most recent tools for genetic manipulation. Even if clinical safety of these tools are still to be clarified and there is undoubtedly still room for the improvement of such approaches, the ability to edit specific genome sequences could revolutionize the whole cell biology, biotechnology, cell engineering and gene therapy areas. Such tools may allow approaches such as add back of gene function, site-directed gene corrections and gene replacements, impacting activities such as animal transgenesis and the incipient logic-systems and biological fields. Hopefully the combination of gene delivery approaches such as those described in this review with the new gene editing tools will turn gene therapy into a more effective and curative approach.

## Additional files

10.1186/s12967-016-1047-x Current clinical trials using lentiviral systems available on the Journal of Gene Medicine database (http://www.abedia.com/wiley/vectors.php).

10.1186/s12967-016-1047-x Current Sleeping beauty transposon’s clinical trials on the Journal of Gene Medicine database (http://www.abedia.com/wiley/vectors.php).

## Abbreviations

ADA: adenosine deaminase
GTAC: Gene Therapy Advisory Committee
CAR: chimeric antigen receptor
CRISPRs: clustered regularly-interspaced short palindromic repeats
CTL: cytotoxic T lymphocyte
GMP: good manufacturing practices
hESCs: human embryonic stem cells
hiPSCs: human induced pluripotent stem cells
HIV: human immunodeficiency virus
HSCs: hematopoietic stem cells
ITRs: inverted terminal repeat sequences
MLV: Moloney leukemia virus
PB: piggyBac
SCID: severe combined immunodeficiency
SB: sleeping beauty
TCR: T cell receptor
TALENs: transcription activator-like effector nucleases
X-SCID: X-linked severe combined immunodeficiency
ZFNs: zinc finger nucleases
