# Supplementary material for: Retroviral vectors and transposons for stable gene therapy: advances, current challenges and perspectives
Source: J Transl Med. 2016 Oct 12;14:288. doi: 10.1186/s12967-016-1047-x (PMC5059932; doi:10.1186/s12967-016-1047-x)
Supplement: Supplementary file 1 — 10.1186/s12967-016-1047-x Current clinical trials using lentiviral systems available on the Journal of Gene Medicine database (http://www.abedia.com/wiley/vectors.php). [file 12967_2016_1047_MOESM1_ESM.doc]

Supplementary table 1. Current clinical trials using lentiviral systems available on the Journal of Gene Medicine database (<http://www.abedia.com/wiley/vectors.php>).

| Disease | Clinical trial ID | Gene | Cell source | Target cells | Clinical Phase | Status |
| --- | --- | --- | --- | --- | --- | --- |
| Brain tumours | AU-0030 | MGMT | - | - | I | open |
|  | US-0898 |  | - | CD34+Cells |  |  |
|  | CN-0041 | EGFR antisense | Autologous | T cells |  |  |
|  | CN-0047 |  |  |  |  |  |
|  | US-1271 | IL-13R scFvFc-Zeta T Cell Receptor 41BB-Costimulatory Chimeric Receptor Truncated CD19 |  |  |  |  |
|  | US-1302 | CD171 Chimeric Antigen Receptors/lgG4hinge-CD28tm; 4-1BB; zeta; T2A; EGFRt (2nd generation) or lgG4hinge-CD28tm-cyto; 4-1BB, zeta; T2A, EGFRt (3rd generation) | Autologous | T cells |  |  |
|  | US-1267 | T Cell Receptor Specific for EGFRvIII | Autologous | Lymphocytes | I | open |
| Blood related cancers | US-1169 | Alpha and Beta Chains of WT1 T Cell Receptor | - | - | I/II | open |
|  | US-1025 | CD19 Antigen Specific-Zeta T Cell Receptor | Autologous | T cells | I |  |
|  | CN-0037 | CD28 CAR; CD137 CAR | - | - |  |  |
|  | CN-0042 | Anti-CD30 | Autologous | T cells | I/II |  |
|  | CN-0048 |  |  |  |  |  |
|  | CN-0043 | Anti-CD19 |  |  |  |  |
|  | CN-0049 |  |  |  |  |  |
|  | US-1300 | CD19 Chimeric Antigen Receptors Expressing Tandem TCR and 4-1BB Costimulatory Domains |  | Tcells | II |  |
|  | US-1330 | NY-ESO-1 | - | DC* | I |  |
|  | US-1291 | CD 19+ Leukemia or Lymphoma | Autologous | Tcells |  |  |
|  | US-1292 | CD19 Antigen Specific Chimeric Antigen Receptor (CAR) Epidermal Growth Factor Receptor |  | Tcm** |  |  |
|  | US-0793 | CD19 Antigen Specific-Zeta T Cell Receptor |  | T cells |  |  |
|  | US-1197 |  |  | - | II |  |
|  | US-1213 | CD19 Antigen Specific CAR EGFR |  | Tcm | I/II |  |
|  | US-1233 |  |  | - |  |  |
|  | US-1320 | CD22 Chimeric Antigen Receptors |  | T cells Expressing CD22 | I |  |
|  | US-0892 |  |  | T cells |  |  |
|  | US-1062 | CD19 Antigen Specific Chimeric Antigen Receptor (CAR) |  |  | I/II |  |
|  | US-1183 | CD19 Antigen Specific Chimeric Antigen Receptor (CAR) -EGFR |  | Tcm | I |  |
|  | US-1250 |  |  |  |  |  |
|  | US-1279 |  |  |  |  |  |
|  | US-1258 | CD19 Antigen Specific-Zeta T Cell Receptor/TCR and 4-1BB Signaling Domains |  | T cells | II |  |
|  | US-1150 | CD19 Antigen Specific Chimeric Antigen Receptor (CAR) | Allogeneic | CD8+ T cells | I/II |  |
|  | US-1161 | CD19 Antigen Specific-Zeta T Cell Receptor | - | - | I |  |
|  | US-1259 | CD19 Antigen Specific-Zeta T Cell Receptor/TCR and 4-1BB Signaling Domains | Autologous | T cells | II |  |
|  | US-1351 | CD19 Chimeric Antigen Receptors Expressing Tandem TCR and 4-1BB Costimulatory Domains |  |  |  |  |
|  | US-1299 |  |  |  |  |  |
|  | US-1287 | CD123 Specific Chimeric Antigen Receptor (CAR) EGFR |  |  | I |  |
|  | US-1056 | High Affinity T Cell Receptor Specific for MAGE-A3 or NY-ESO-1 |  |  |  |  |
|  | US-1338 | LVsh5; C46 | - | H. Cells | I/II |  |
| Synovial Sarcoma | US-1071 | High Affinity T Cell Receptor Specific for NY-ESO-1 | Autologous | Tcells | I | open |
| Oesophagogastric Cancer | UK-0207 | NY-ESO-1 | Autologous | T cells | II | open |
| Lung cancer | UK-0221 | Tumor necrosis factor-related apoptosis-inducing ligand (TRAIL) | - | Stem cells | I/II | open |
| Stage III-b or IV Non-Small Cell Lung Cancer | US-1356 | NY-ESO-1/LAGE-1 | Autologous | CD4+ and CD8+ T cells | I/II | open |
| Malignant melanoma | US-1057 | High Affinity T Cell Receptor Specific for MAGE-A3 or NY-ESO-1 | - | - | I | open |
|  | US-0791 | Alpha and beta chains of T-cell receptor specific for MART-1 | Autologous | T cells | I | open |
|  | US-1086 | Alpha and Beta T Cell Receptor Specific for Tyrosinase | Autologous | T cells | I | open |
|  | US-1112 |  |  |  |  |  |
| Ovarian cancer | US-1076 | Alpha-folate Receptor-scFv with Signaling Domains Comprised of TCR-zeta, and 4-1BB | Autologous | T cells | I | open |
|  | [US-0980](http://www.abedia.com/wiley/record_detail.php?ID=1771) | A-mesothelin-scFv with Signaling Domains Comprised of TCR-zeta, CD28, and 4-1BB |  |  | I | open |
| Hepatocellular Carcinoma | US-1176 | NY-ESO-1; LAGE-1 | Autologous | CD4+ and CD8+ T cells | I/II | open |
|  | US-1177 |  |  |  |  |  |
| Metastatic Expressing NY-ESO-1 | US-1266 | NY-ESO-1 | - | DC | I | open |
|  | US-1344 | NY-ESO-1 | - | DC | I | open |
| Mesothelin Expressing Cancer | US-1277 | mesothelin-scFv with Signaling Domains Comprised of TCR , CD28, and 4-1BB | Autologous | T cells | I | open |
| Metastatic Pancreatic | US-1352 | Humanized CD19-scFv with Signaling Domains Comprised of TCR , CD28, and 4-1BB-mesothelin-scFv with Signaling Domains Comprised of TCR , CD28, and 4-1BB | Autologous | T cells | I | open |
| Breast cancers | US-1378 | T Cell Receptor alpha and beta cDNAs Chains | Autologous | T cells | I/II | U.R*** |
| HIV infection | BE-0026 | HIV-1 Gag-Pol-Nef | - | - | I/II | open |
|  | US-0488 | HIV-1 Env | Autologous | CD4+ cells | I | closed |
|  | US-0875 |  |  |  | II | open |
|  | US-0646 | Antisense env |  |  | I/II |  |
|  | US-0667 | VRX496 |  |  |  |  |
|  | US-1237 | RNAi Targeted at HIV tat and ver; RNA Decoy for TAR; Ribozyme Targeted at CCR5 Cytokine Receptor (CCR5RZ) |  | CD34+ Cells | I |  |
|  | US-1296 |  |  |  |  |  |
|  | US-0725 |  |  |  |  |  |
|  | US-0737 | RNAi Targeted at HIV tat and ver; RNA Decoy for TAR; Ribozyme Targeted at CCR5 Cytokine Receptor (CCR5RZ); HIV-1 Env |  |  |  |  |
|  | US-0871 |  |  | CD4+ cells | I/II |  |
|  | US-0921 | T Cell Receptor alpha and beta Chain specific for HIV-Gag |  | T cells | I |  |
|  | US-0975 | HIV-1HXB2 gag, truncated pol, vpr, rev, tat, nef, and Envelope Proteins | - | - |  |  |
|  | US-1130 | HIV genes; C46 | - | - | I/II |  |
|  | US-1308 | CCR5shRNA/RNA Decoy for TAR/TRIM5alpha | Autologous | CD34+ Cells |  |  |
|  | US-1153 |  |  |  |  |  |
|  | US-1337 | CCR5shRNA; C46; P140K |  |  | I |  |
| Syndrome (WAS)  Wiskott Aldrich | FR-0047 | WAS | - | HSCs | I/II | open |
|  | FR-0062 |  | autologous | CD34+ cells |  |  |
|  | FR-0063 |  |  |  |  |  |
|  | UK-0168 |  | - | - | I |  |
|  | US-1052 |  | Autologous | CD34+ Cells B.M |  |  |
| X-linked chronic granulomatous disease (G1XCGD) | FR-0067 | gp91phox | Autologous | CD34+ cells | I/II | open |
|  | XX-0029 |  |  |  |  |  |
| X-Linked Severe Combined Immune Deficiency | UK-0198 |  |  |  |  |  |
|  | US-0963 | Gamma c common chain receptor | Autologous | CD34+ cells | I | open |
|  | US-0964 |  |  |  |  |  |
| Hemoglobinopathies (Sickle Cell Anemia and Thalassemia Major) | FR-0029 | Human -Globin | - | - | I/II | open |
|  | FR-0055 |  | - | - |  |  |
|  | IT-0026 |  | - | - |  |  |
|  | US-1187 |  | Autologous | CD34+ Cells |  |  |
|  | US-0852 |  |  |  | I |  |
|  | US-1023 |  |  |  |  |  |
|  | US-1067 |  |  |  |  |  |
|  | US-1164 |  |  |  |  |  |
|  | US-1254 |  |  |  |  |  |
| Fanconi Anaemia | XX-0020 | FANCA | Autologous | CD34+ cells | I/II | open |
|  | US-0895 |  |  |  | I |  |
|  | US-1105 |  |  |  |  |  |
| Adenosine deaminase (ADA) deficiency | UK-0199 | Adenosine deaminase (ADA) | Autologous | CD34+ cells | I/II | open |
|  | US-1006 |  |  |  |  |  |
| Metachromatic Leukodystrophy and adrenoleukodystrophy | CN-0038 | - | - | - | I/II | open |
|  | CN-0044 | - | - | - |  |  |
|  | IT-0019 | Arylsulfatase A | - | - |  |  |
| X-linked ALD | FR-0028 | ABCD-1 Gene (ALD protein) | Autologous | CD34+ cells | I/II | closed |
| AdenoleuKodystrophy | FR-0042 |  | - | - | III | open |
| Childhood cerebral adrenoleukodystrophy | FR-0066 |  | Autologous | CD34+ cells | II/III | open |
|  | US-1073 |  |  |  | I/II |  |
|  |  |  |  |  |  |  |
| Parkinson?s disease | FR-0041 | Tyrosinase GTP-cyclohydrolase 1 Dopa decarboxylase | - | - | I/II | open |
| Netherton syndrome | UK-0191 | Dopa decarboxylase; Tyrosine hydroxylase ;GTP-cyclohydrolase 1 | - | - | I/II | open |
|  | UK-0201 |  | - | - |  | U.R |
| Mucopolysaccharidosis Type VII (MPS VII) | US-0758 | SPINK5 | - | - | I | open |
| Age-related Macular Degeneration (AMD) | US-1061 | Beta-Glucuronidase | Autologous | CD34+ cells | I | open |
| Stargardt`s Macular Degeneration (SMD) | FR-0048 | Retina-Specific ABC Transporter (ABCR) | - | - | I | open |
|  | US-1085 |  | - | - | I/II |  |
| Retinitis Pigmentosa Associated with Usher Syndrome Type 1B | FR-0060 | MYO 7A | - | - | I/II | open |
|  | US-1102 |  | - | - |  |  |
| Chronic Granulomatous Disease | [US-1223](http://www.abedia.com/wiley/record_detail.php?ID=2014) | gp91phox | Autologous | CD34+ B.M cells | I/II | open |
|  | XX-0025 |  | - | CD34+ cells |  |  |
| Recessive Dystrophic Epidermolysis Bullosa (RDEB) | US-1370 | Human collagen type 7 A1 | - | Fibroblasts | I/II | open |
| Peripheral Artery Disease | US-1375 | Vascular Endothelial Growth Factor (VEGF165) | Allogeneic | MSCs | I | open |
| Hemophilia A | US-1112 | Factor VIII | Autologous | CD34+ Cells | I | open |

DC*: Dendritic cells

Tcm**: Central Memory T Lymphocytes

U.R***: Under Review
