# Supplementary material for: Retroviral vectors and transposons for stable gene therapy: advances, current challenges and perspectives
Source: J Transl Med. 2016 Oct 12;14:288. doi: 10.1186/s12967-016-1047-x (PMC5059932; doi:10.1186/s12967-016-1047-x)
Supplement: Supplementary file 2 — 10.1186/s12967-016-1047-x Current Sleeping beauty transposon’s clinical trials on the Journal of Gene Medicine database (http://www.abedia.com/wiley/vectors.php). [file 12967_2016_1047_MOESM2_ESM.doc]

Supplementary table 2. Current Sleeping beauty transposon`s clinical trials on the Journal of Gene Medicine database (<http://www.abedia.com/wiley/vectors.php>).

| Disease | Clinical trial ID | Gene | Cell source | Target cells | Clinical Phase | Status |
| --- | --- | --- | --- | --- | --- | --- |
| B-cell malignancies | US-0922 | CD19 Antigen Specific-Zeta T Cell Receptor | Autologous | T Lymphocytes | I | open |
|  | US-1003 | CD19 Antigen Specific-Zeta T Cell Receptor | Allogeneic | HLA Matched T Lymphocytes |  |  |
|  | US-1022 |  |  | Umbilical Cord Blood-derived Lymphocytes |  |  |
|  | US-1142 |  | Autologous | CD4+ and CD8+ T lymphocytes |  |  |
|  | US-1192 |  |  |  |  |  |
|  | US-1203 |  |  |  |  |  |
|  | US-1225 |  |  |  |  |  |
|  | US-1236 |  | Allogeneic | Umbilical Cord Blood-derived Lymphocytes |  |  |
|  | US-1353 | CD19 Antigen Specific-Zeta T Cell Receptor ; Interleukin-15 (IL-15) | Autologous | Primary CD3+ Lymphocytes |  |  |
| Metastatic Breast cancer | US-1360 | Murine MUC1 Chimeric Antigen Receptor CD28/CD3 /OX40 Caspase 9, Interleukin-12 (IL-12) | Autologous | T Lymphocytes | I/II | U.R* |

U.R*: Under review
